# Bone metabolism and evolutionary origin of osteocytes: Novel application of FIB-SEM tomography

**DOI:** 10.1126/sciadv.abb9113

**Published:** 2021-03-31

**Authors:** Yara Haridy, Markus Osenberg, André Hilger, Ingo Manke, Donald Davesne, Florian Witzmann

**Affiliations:** 1 Museum für Naturkunde, Leibniz-Institut für Evolutions- und Biodiversitätsforschung, Berlin, Germany.; 2 Helmholtz Centre for Materials and Energy (HZB), Hahn-Meitner-Platz 1, 14109 Berlin, Germany.; 3 Department of Earth Sciences, University of Oxford, OX1 3AN Oxford, UK.; 4 Institut de Systématique, Évolution, Biodiversité (UMR 7205), Muséum National d’Histoire Naturelle, CNRS, Sorbonne Université, EPHE, 75005 Paris, France.

## Abstract

Lacunae and canaliculi spaces of osteocytes are remarkably well preserved in fossilized bone and serve as an established proxy for bone cells. The earliest bone in the fossil record is acellular (anosteocytic), followed by cellular (osteocytic) bone in the jawless relatives of jawed vertebrates, the osteostracans, about 400 million years ago. Virtually nothing is known about the physiological pressures that would have initially favored osteocytic over anosteocytic bone. We apply focused ion beam–scanning electron microscopy tomography combined with machine learning for cell detection and segmentation to image fossil cell spaces. Novel three-dimensional high-resolution images reveal areas of low density around osteocyte lacunae and their canaliculi in osteostracan bone. This provides evidence for demineralization that would have occurred in vivo as part of osteocytic osteolysis, a mechanism of mineral homeostasis, supporting the hypothesis that a physiological demand for phosphorus was the principal driver in the initial evolution of osteocytic bone.

## Teaser

New applications of imaging technology reveal that 425 million–year–old fossil bone cells had modern mineral metabolism abilities.

## INTRODUCTION

**Fig. 1:**
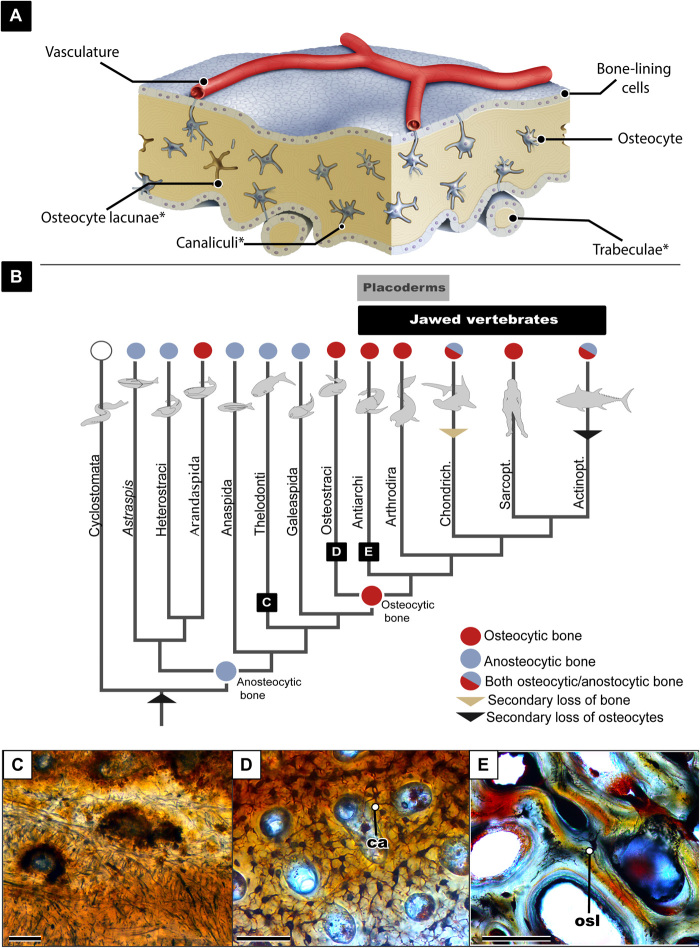
Overview of the evolution of osteocytes. (**A**) Schematic drawing showing bone microanatomy; *denotes the bone anatomy that is readily preserved in fossil material. (**B**) Vertebrate phylogeny showing the evolution of osteocytic versus anosteocytic bone modified from (*38*). (**C**) Image of histological section of fossilized anosteocytic bone of a heterostracan (MB.f.TS.2302) showing tubules but no lacunae present. (**D**) Histological section of fossilized osteocytic bone of an the osteostracan *Tremataspis mammillata* (MB.f.TS.463) showing dense osteocyte lacunae and canaliculi. (**E**) Histological section of fossilized placoderm bone showing both osteocyte lacunae and osteonal remodeling. osl, osteocyte lacunae; ca, canaliculi. All scale bars are 100 μm.

Bone is an essential innovation for all vertebrate life and can undoubtedly be credited for the immense diversity of vertebrate lifestyles; from swimming, to walking, to flying, bone is the material that has provided a literal scaffolding for evolutionary diversity. Despite the significance of bone, unexpectedly little is known about its evolutionary origin. It is impossible to understand bone without considering the building blocks that make it a living lattice: the mineral, the protein, and the cellular components. The cellular components of bone have been well studied in modern humans in hopes of understanding how to better heal and grow this regenerative tissue (*1*, *2*). While the developmental relationships of the four cell types (osteoprogenitor, osteoblasts, osteocytes, and osteoclasts) in modern bone are well known (*2*, *3*), very little is known about the evolutionary origins of these cells. Here, we focus on osteocytes and their processes: Osteocytes are mature osteoblasts that become entrapped within lacunae in the mineral matrix of bone, the osteocytes’ cytoplasmic processes are contained in canaliculi, and, together, the osteocyte and these processes are what compose the intricate lacunocanalicular network (LCN), giving bone the interconnectivity similar to that of the neural network (*2*). Several studies have attributed various physiological roles to osteocytes, including bone remodeling, mechano-sensation, and mineral homeostasis [see (*2*, *3*) and references therein]. Osteocytes are uniquely important in the eyes of paleontologists, as they are the only cells to reliably preserve their shape in fossils through their cell lacunae (Fig. 1A). The cell lacunae within bone conform so readily to osteocyte shape that the size of these lacunae has been used to estimate genome sizes (*5*, *6*). In addition, the density of these lacunae has been used as a proxy for metabolism (*7*–*9*), and the shape of the lacunae walls can inform us about mineral homeostasis (*10*–*12*). Thus, while the actual cell does not preserve in fossils, the lacuna acts as an established model for studying cellular structures in extinct vertebrates.

**Fig. 2:**
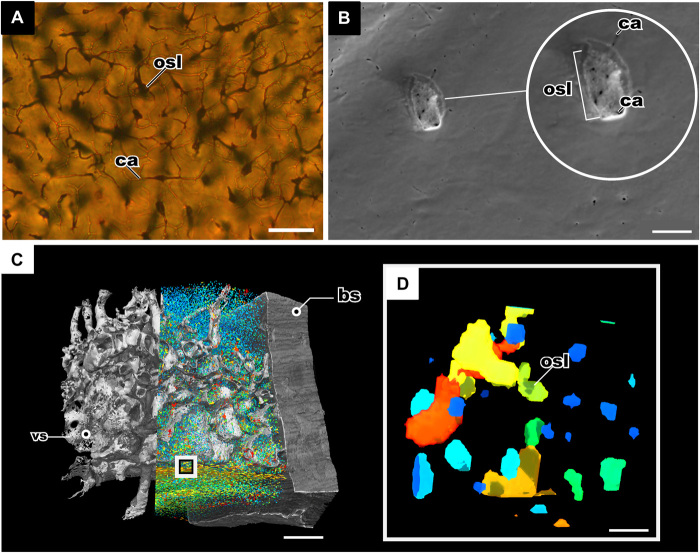
Comparing the state-of-the-art imaging technology of fossil osteocyte lacunae. (**A**) Histological thin section of bone in the osteostracan *T. mammillata* (MB.f.TS.463), imaged with transmitting light microscopy showing osteocyte lacunae and canaliculi; scale bar, 100 μm. (**B**) SEM of the etched surface of the dermal bone of *Bothriolepis trautscholdi* (MB.f.9188a) produced this image of the internal surface of the fossil osteocyte lacunae, and the small black holes represent the emanating canaliculi; scale bar, 10 μm. (**C**) Synchrotron tomography of bone of *B. trautscholdi* (MB.f.9188a) with the vasculature and osteocytes segmented; scale bar, 0.4 mm. (**D**) Close-up of tomography in (C) showing the resolution of the osteocyte lacunae volumes; scale bar, 10 μm. bs, bone sample; vs, vasculature channels.

Osteocyte lacunae in fossilized bone were first illustrated in the mid-19th century during the course of paleohistological studies [e.g., (*13*–*15*)]. These first investigations of fossil bone microstructure were done through the use of histological thin sections and transmitted light microscopy, which has been the traditional method for studying fossil osteocyte lacunae and their canalicular processes (*7*, *16*–*19*). Pawlicki (*20*) described in depth the morphology of osteocytes in Late Cretaceous dinosaur bones from Mongolia based on classical light microscopical investigations but also applied new methods like transmission electron microscopy (TEM) and scanning electron microscopy (SEM) to illustrate the morphology of the lacunae and canaliculi in detail. However, despite the high-resolution quality of the TEM and SEM images, the visualizations are two-dimensional (2D) and are thus less informative concerning details of spatial arrangement and connectivity of the cell lacunae. Synchrotron micro–computed tomography (μCT) studies carried out on early fossil vertebrates and modern taxa (*18*, *19*, *21*, *22*) have enhanced our knowledge (*18*, *19*, *21*) about the density, spatial organization, and arrangement of bone cell lacunae in fossil bone as well as the lacunar volume. However, even the highest-resolution scans (0.678 μm voxel size) are usually not able to show the morphology and arrangement of the canaliculi in detail. In the few cases where canaliculi are visible from synchrotron μCT, resolution is not sufficient to illustrate their connections, branching, and spatial arrangement in enough detail (*21*). In Fig. 2, we compare the state of the art in fossil osteocyte lacunae imaging technology.

The origin of the vertebrate mineralized skeleton phylogenetically and temporally predates the origin of gnathostomes (jawed vertebrates), rendering early jawless vertebrates essential for understanding the earliest bone physiology (Fig. 1B). Osteostracans are a group of early jawless vertebrates ranging from the middle Silurian to the Late Devonian (433 to 372 million years ago) (*23*–*25*). Osteostracans are the sister-group to gnathostomes, and their last common ancestor was the first to evolve osteocytes. The earliest jawed vertebrates, the paraphyletic “placoderms,” appear in the early Silurian more than 430 million years ago (*26*), and their last common ancestor with modern gnathostomes was the first to evolve secondary osteons (*27*, *28*). Osteostracans and placoderms are therefore of particular interest in this study, as they document two major developments in bone evolution. Last, in this study, we focus on dermal bone rather than endochondral bone from both taxa as it is more easily accessible because of its superficial location; it is possible that endochondral bone may have different properties on a cellular level. Endochondral bone in skull elements has been recently reported in the “placoderm” *Minjinia turgenensis* (*29*) but has yet to be reported in the skull material of any Osteostracan.

To understand cellular bone, one must come to terms with the intriguing fact that not all bone is cellular; in fact, more than half of modern teleost fishes have acellular bone, also known as anosteocytic bone (*30*–*35*), which lacks osteocytes but maintains the other three types of bone cells. Furthermore, the earliest vertebrates with bone (e.g., heterostracans) show a peculiar type of anosteocytic bone, called aspidin (*36*–*39*). Osteocytes appeared in the osteostracan-gnathostome clade, and possibly (but controversially) independently in another lineage of jawless vertebrates, arandaspids (Fig. 1) (*40*).Thus, it is particularly intriguing that the first bone to appear in vertebrate evolution lacks osteocytes (Fig. 1) (*36*–*39*). Consequently, this leads to the following questions: (i) What selective pressures caused the initial evolution of osteocytic bone; (ii) what physiological advantage did osteocytes provide to early bony vertebrates?

To test whether osteocytic bone confers selective advantages over anosteocytic bone, one should examine the roles that osteocytes have been credited with and then examine whether those same roles exist in anosteocytic bone. Osteocytes have been credited with three main roles: (i) orchestrating bone remodeling, (ii) mechano-sensing, and (iii) mineral homeostasis (*1*, *4*). Recent studies have demonstrated that anosteocytic bone of modern teleosts displays signs of remodeling and mechano-sensing (*4*, *31*, *41*–*43*). Therefore, two of the main roles of osteocytes in bone physiology (i.e., remodeling and mechano-sensing) can also be performed by anosteocytic bone. While this suggests that the first anosteocytic bone in jawless vertebrates also had these capabilities, the fact that osteocytes are not the exclusive pathways for the fulfillment of these physiological needs renders it unlikely that these functions were the driving force behind the initial evolution of osteocytic bone. This leaves mineral metabolism via osteocytic osteolysis as the final osteocyte role that may have conferred a major advantage to osteocytic bone (*4*, *31*, *42*, *43*). Osteocytic osteolysis is a localized process of matrix removal and is often followed by redeposition in the lacunar wall as a mechanism of mineral homeostasis (*12*, *44*, *45*). Mineral metabolism via osteocytic osteolysis is inherently impossible in anosteocytic bone, as it lacks osteocytes, and various studies in modern teleosts have discussed how the inability to perform this physiological process may be a disadvantage in times of phosphate deficiency (*4*, *31*, *42*, *43*, *46*, *47*). Therefore, it may be presumed that this was also the case in the earliest jawless vertebrates with anosteocytic bone. However, the subsequent evolution of osteocytes and the ability for osteocytic osteolysis in extant vertebrates does not necessarily imply that the earliest osteocytes in osteostracans and gnathostomes had this capability. As previously stated, very little is known about the morphology of the first osteocytes, which is due to the inherent limitations of the fossil record and of imaging technology.

**Fig. 3:**
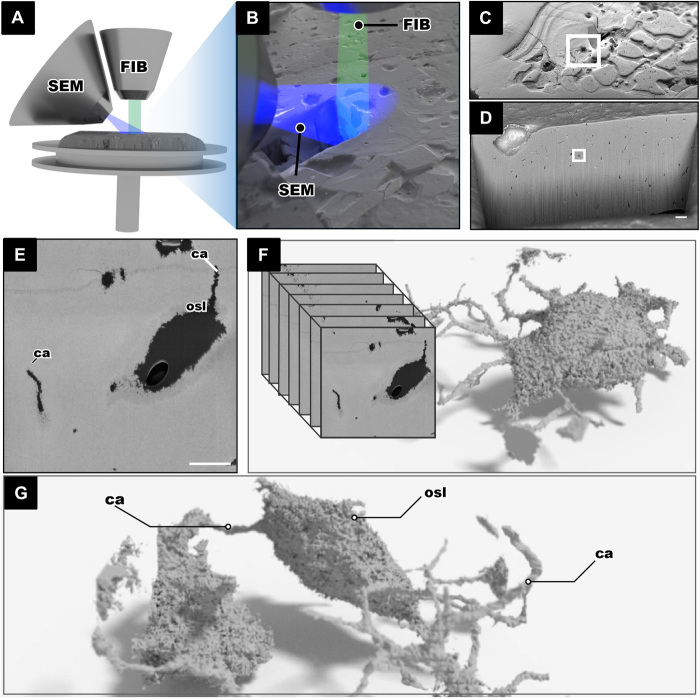
FIB-SEM tomography imaging and processing of the fossil jawless vertebrate *T. mammillata* (MB.f.9025). (**A** and **B**) FIB-SEM setup showing the FIB in relation to the SEM both aimed at the region of interest. (**C**) Bone surface with an excavate area made by the FIB. (**D**) Internal wall of the excavated area lined with small black dots that are the fossil osteocyte lacunae. (**E**) Single osteocyte lacuna from the surface that is scanned; the single SEM image shows the lacunae and canaliculi in black and the mineralized bone in gray; scale bar, 5 μm. (**F** and **G**) An image stack is obtained, and 3D made of fossil LCN can be made.

The only previous study on osteocytic osteolysis in fossil bone is that of Bélanger (*10*) in an indeterminate Cretaceous reptile, using classical light microscopic investigation that identifies osteocytic osteolysis based on uneven edges and larger than average lacunae. However, we believe that the criteria for identifying osteocytic osteolysis remain difficult to assess in a conventional histological slide. To test for osteocytic osteolysis in fossils using 3D data sources, the osteocyte lacunae must be imaged at a resolution below 100 nm to identify evidence of resorption, dissolution, or uneven edges. In previous studies, this was hampered by the limitations of resolution of 3D imaging that could be achieved with fossilized material. In this study, it has been made possible with focused ion beam (FIB) tomography, an imaging technique mainly used for 3D investigations in material science (e.g., battery materials, alloys, thin films, and semiconductors) (*48*) and under cryo-conditions in biology (*49*). FIB-SEM has been previously applied to image fossil material in 2D (*50*, *51*) and, to our knowledge, only in 3D in microfossils (*52*, *53*). Last, FIB-SEM has been used sparingly, but promisingly, to study osteocyte lacunae in modern bone (*54*, *55*) and is applied in the present study to image fossil cell spaces (Fig. 3).

Here, we aim to (i) describe the morphology of the earliest osteocytes in jawless vertebrates (osteostracans) and early gnathostomes (placoderms); (ii) explore whether there is evidence that the earliest osteocytes were capable of osteocytic osteolysis, and whether mineral homeostasis was a possible driving force for initial evolution of cellular bone; (iii) explore the relationship between size and interconnectivity of fossil osteocytes; and (iv) establish a protocol for the use of FIB for fossil bone material. Through a combination of recent discoveries in bone physiology and new applied technologies, new hypotheses for the origin of cellular bone can be examined.

## RESULTS

### Osteocyte lacunae

In general, the isolated osteocyte lacunae and the associated canalicular network in both samples are reminiscent of their modern counterparts in shape and distribution.

#### Tremataspis mammillata (Osteostraci) MB.f.9025 (Late Silurian; ~423 to 428 million years ago)

**Fig. 4:**
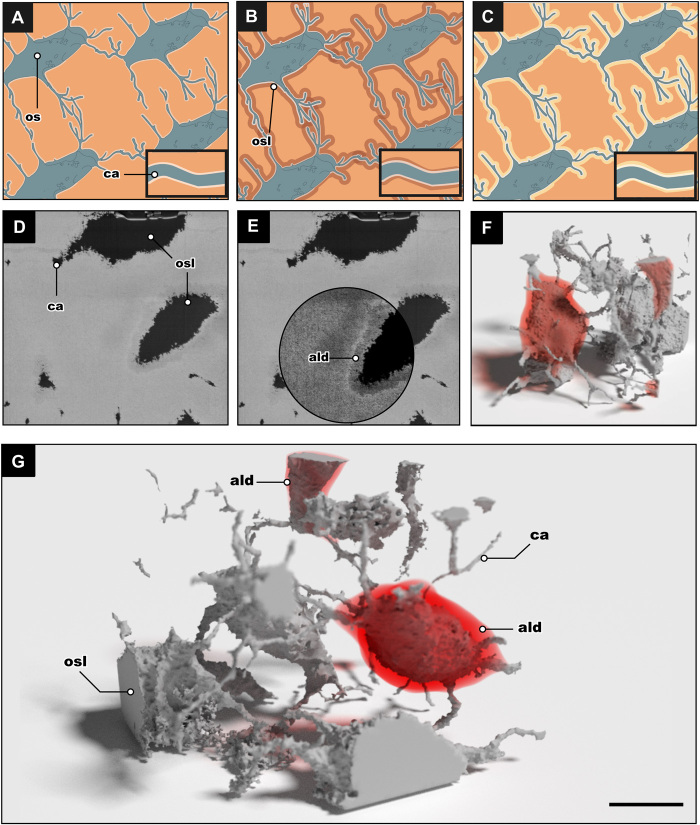
Osteocytic osteolysis as a mechanism for early mineral metabolism. (**A** to **C**) Illustrations depicting the process of osteocytic osteolysis; the phases are stasis phase, dissolution phase, and redeposition phase, respectively. (**D**) Single SEM image from the FIB-SEM acquisition showing the air-filled osteocyte lacunae and canaliculi of *T. mammillata*. (**E**) Same SEM image as in (D) with contrast shifted to show the demineralized zone surrounding the lacunae. (**F** and **G**) 3D render of the stack of images from (D and E) *T. mammillata* (MB.f.9025). The 3D model shows several osteocytes and their canaliculi, with the red areas showing where the “areas of low density” were found. ald, area of low density; os, osteocyte. Scale bar, 5 μm.

In the osteostracan specimen, a fragment of the anterior region of the headshield was analyzed where nine cell lacunae were virtually isolated and then visualized, of which seven are incomplete due to the field of view (31 μm × 24 μm × 23 μm; 17,112 μm^3^). The total volume of the pores in the bone structure was 1398 μm^3^, which represents the total volume of all lacunae and canaliculi; thus, the volume of actual bone is 15,430 μm^3^. The two complete cell lacunae have a volume of 475.5 μm^3^. The lacunae vary from ovate to round in shape, which can be seen in the traditional thin sections of this taxon (Figs. 1D and 2A) and which is now corroborated by the new 3D FIB-SEM tomography. The two complete lacunae isolated through the FIB-SEM tomography are ovate and flattened in shape, with rough uneven borders. However, roughness in the borders is found in all of the isolated cell lacunae in both samples and is likely due to the use of FIB during the method of acquisition. What cannot be attributed to the method of acquisition is an area of low-signal intensity. This structurally altered area surrounding the lacunae is interpreted as an area of low density (ALD). ALD is found surrounding one complete lacuna, two partial lacunae just out of frame, and particular canalicular junctions. ALD appears as a dark halo around the lacunae, which contrasts with the grayscale colors of the lacunae (black) and the fossilized bone (gray) (Fig. 4). When isolated, this halo completely surrounds one of the lacunae and the two partially visible lacunae. These areas of low density were not found on directly neighboring lacunae and were not continuous with the length of the canaliculi and therefore are not thought to be taphonomic alterations to the fossil bone. The one complete osteocyte lacuna with an ALD was further analyzed; the volume of the lacunae is 232 μm^3^, and the volume of the isolated surrounding low-density region is 368 μm^3^. Therefore, the total volume of these two regions together is more than twice the volume of the original lacuna.

#### Bothriolepis trautscholdi (Antiarchi, “Placodermi”), MB.f.9188 (Late Devonian, ~372 to 382 million years ago)

**Fig. 5:**
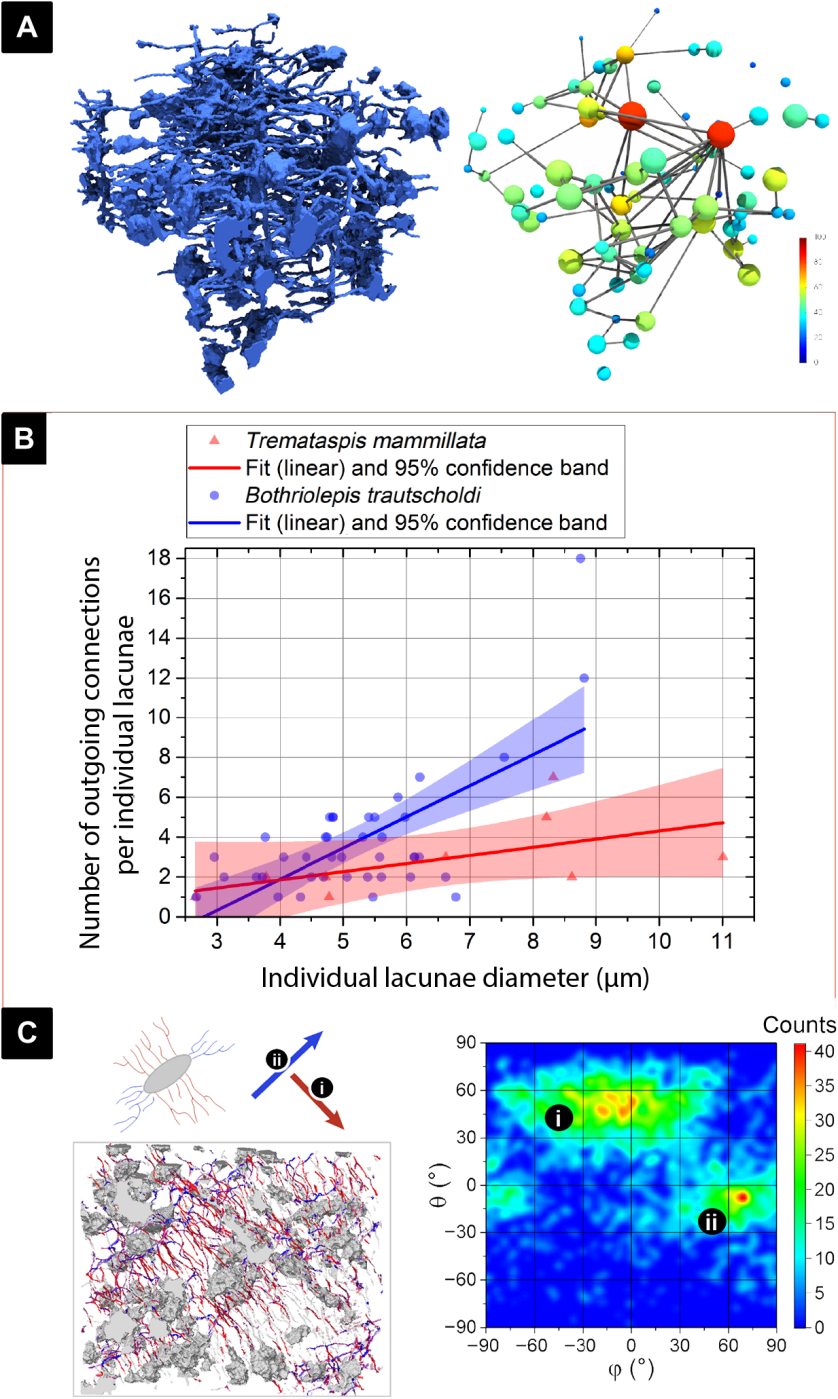
Analysis of osteocyte lacunae size in relation to canalicular number and direction. (**A**) The 3D cell spaces and canaliculi of the *B. trautscholdi* (MB.f.9188a) sample were reduced to skeletal forms (see data analysis in Materials and Methods) to visually illustrate that larger cell lacunae (in warmer colors) have more canaliculi connections. (**B**) Relationship between cell lacunae size and number of outgoing canaliculi in both tested samples, *B. trautscholdi* (MB.f.9188a) and *T. mammillata* (MB.f.9025), showing that the larger the cell, the more outgoing canaliculi emanate from it (related analysis parameters are shown in table S2A). (**C**) Directionality analysis on the *B. trautscholdi* (MB.f.9188a) specimen shows that there are two predominant directions where canaliculi emanate from the osteocyte lacunae, and that these two directions are almost perpendicular to one another as shown in the heatmap.

The second specimen is a fragment of the anterior headshield of the antiarch placoderm *Bothriolepis*, a representative of the earliest jawed vertebrates and the first known taxon with definite evidence of organized osteonal remodeling (*27*, *37*). The field of view in this specimen was 55 μm × 41 μm × 43 μm (97,965 μm^3^), and a total of 67 osteocyte lacunae were segmented; 27 of the lacunae were partially incomplete as they were close to the border of the tomography. There was a large discrepancy between lacunae size in this sample, with the largest lacunae having a volume of 245.0 μm^3^, similar to the first specimen, and the smallest lacunae having a volume of 3.7 μm^3^. The largest lacunae were less frequent in this sample; only two lacunae had a cell diameter greater than 7 μm (maximum of 8 μm), with most lacunae having a diameter of 4 μm. The smallest diameter was 2 μm (Fig. 5). We recognize that this disparity in lacunae sizes has never been observed before; however, because of the interconnected nature of their canaliculi and their organic shape, we conclude that they are true spaces and not cracks or taphonomic defects. Some of the smallest cell spaces may be canalicular junctions; this would result in an overestimation of cell space density and an underestimation of the average emanating canaliculi as junctions tend to have less canaliculi emanating from them. It is difficult to quantify the junctions from the true lacunae as the data are continuous between the large junctions and the small cell spaces. There were no areas of low bone density observed in this sample; 3.9% of the measured volume consisted of the LCN.

### Canaliculi and interconnectivity

Figures 4 and 5 collectively illustrate the results from several analyses that we performed to better understand the interconnectivity of the LCN. The *Tremataspis* dataset contains only nine osteocyte lacunae, of which only two are complete, and therefore, the analysis of interconnectivity is likely not a true representation of the entire organism. Nevertheless, it is included here for comparison. The *Bothriolepis* dataset is also not likely representative of the entire organism’s skeleton; however, it is a much more robust analysis of 40 osteocyte lacunae and their associated canaliculi. The interconnectivity analysis to our knowledge is the first of its kind. It aims to quantify the connections osteocyte lacunae have with one another via their canaliculi and to see whether there are patterns in connectivity related to the size or distribution of lacunae. We quantified this network by determining the number and direction of canaliculi coming out of each lacuna and the complexity of branching of each canaliculus. This is only a partial representation of true interconnectivity, as we cannot yet trace whether each canaliculus truly terminates in a connection with another cell lacuna or with the bone surface.

A linear regression shows that there is a partly positive correlation between lacuna size and the number of canaliculi that emanate from the lacunae (Fig. 5B and table S2A). With 1.56 ± 0.28 outgoing channels per micrometer lacuna diameter (*R*^2^ = 0.44; *P* < 0.001), the slope is significantly different from zero in the *Bothriolepis* sample. But with 0.41 ± 0.22 outgoing channels per micrometer lacuna diameter (*R*^2^ = 0.32; *P* = 0.11), however, the slope in the *Tremataspis* specimen is not significantly different from zero (using a significance level of 0.05). Comparing both species, there is a significant difference of their individual relation between lacunae size and cell diameter (origin two-sample *t* test: *P* < 0.001). In the *Bothriolepis* sample, the largest lacuna (diameter of 9 μm) had 18 canaliculi emanating from it, while the smallest lacuna (diameter of 2 μm) had 1 to 3 canaliculi emanating from it (Fig. 5B). The *Bothriolepis* sample also allowed a directionality analysis of the canaliculi, and we found that there are two main orientations angled at almost 90° to one another displayed as a heatmap showing frequency of each angle (Fig. 5C). This perpendicular bidirectionality of the canaliculi is known in modern vertebrate taxa, particularly in lamellar bone and around haversian canals (*56*).

**Fig. 6:**
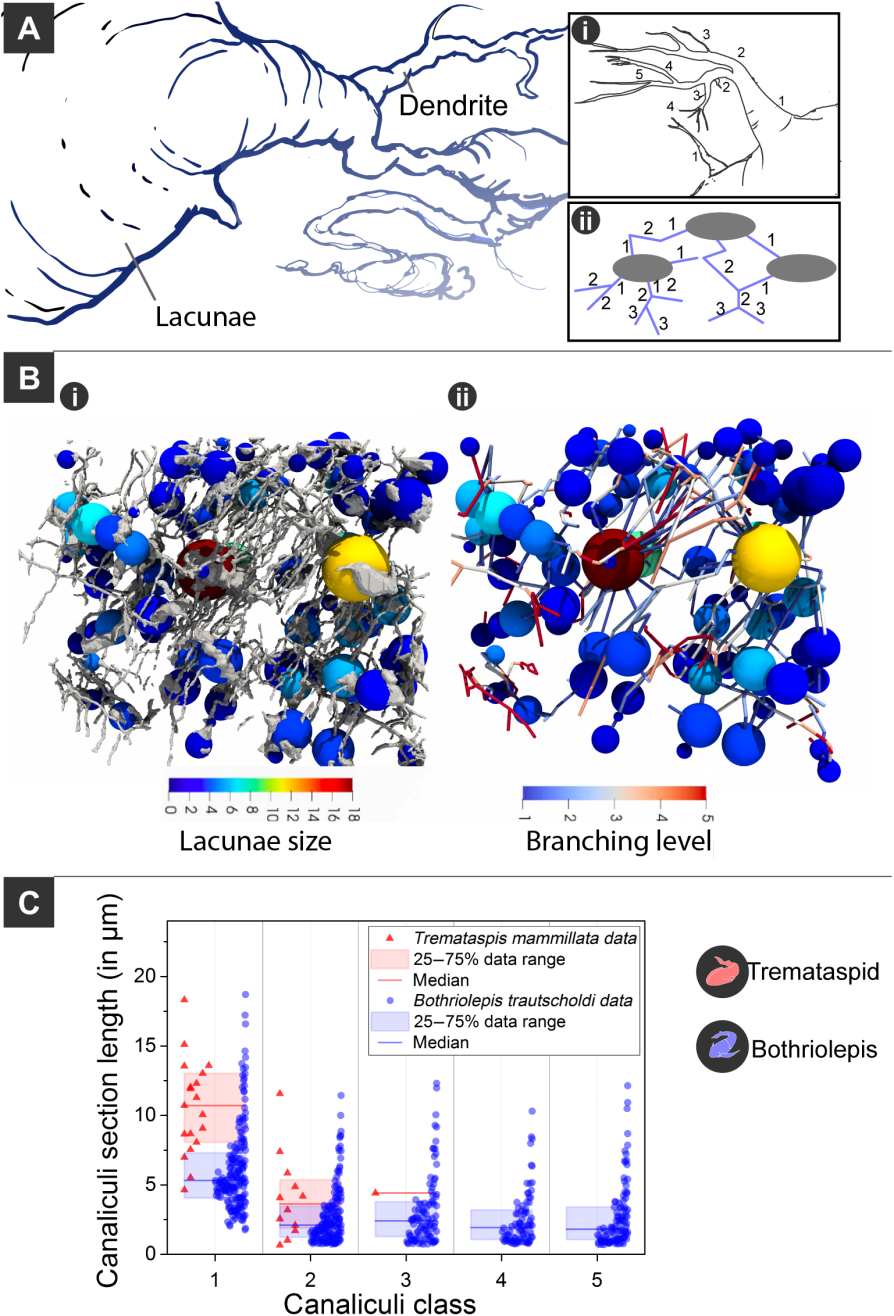
Complexity of canalicular branching in early fossil vertebrates. (**A**) Illustration showing canaliculi emanating from cell body. (i and ii) Illustrations showing the branching classifications. (**B**) (i) *B. trautscholdi* model with spheres scaled to the equivalent cell body radius and colored depending on their outgoing number of canaliculi; (ii) bare network data with the canaliculi section colored depending on their branching class/level. (**C**) Comparative analysis of canalicular branching and length analysis between *B. trautscholdi* (MB.f.9188a) and *T. mammillata* (MB.f.9025).

The degree of canalicular branching also contributes to network complexity and interconnectivity. The canaliculi in *Tremataspis* have been recorded to branch up to three times, whereas the canaliculi in *Bothriolepis* have been recorded to branch up to five times (Fig. 6C), the latter value having also been reported in human osteocytes (*2*). Following the classifications used by Buenzli and Sims (*2*), the first canaliculi to branch from the lacunae was classified as “class 1” and canaliculi branching from class 1 became “class 2” and so forth (Fig. 5A). In both specimens, class 1 canaliculi were the most abundant and the longest followed by class 2 canaliculi (Fig. 6C). This is not unusual as the existence of a higher class of canaliculi is conditional on the presence of the precursor class. However, it is still noteworthy as this is the condition of both specimens and is also characteristic of human canaliculi (*2*). The diameters of the canaliculi are logarithmic normal distributed (fig. S1G; see table S1A for more details). The *Bothriolepis* sample canaliculi have a median diameter of 189.0 ± 5.4 nm, and the median diameter of canaliculi in the *Tremataspis* specimen is 279.2 ± 6.8 nm.

## DISCUSSION

Our results demonstrate that the earliest known osteocytes in the fossil record had similar morphology and likely similar physiological capabilities to their modern counterparts as indicated by their lacunae and canaliculi. In this study, we presented a new method for imaging fossil osteocyte lacunae via FIB-SEM tomography, thereby providing the highest-resolution 3D imaging of the earliest osteocyte lacunae and canaliculi. Through this method, we were able to visualize and quantify the LCN by analyzing the shape, number, direction, and extent of branching per canalicular process. Most features of the LCN are comparable to those of modern osteocytes. However, the most interesting finding is the indirect evidence for the earliest mineral metabolism via osteocytic osteolysis (Fig. 4).

This study aimed to further understand early osteocytes by collecting data on the size and morphology of their lacunae, and the branching and orientation of their canaliculi. We found that the volume of the osteocyte lacunae varied within and between samples. This is not unusual, as several studies have found that cell volume can fluctuate intra- and interspecifically (*3*, *18*, *19*, *21*). The morphology of the lacunae in *Tremataspis* is typical “spindle-shaped” cells that have been documented in 3D in teleosts (*21*), chick (*57*), mouse (*58*), and human osteocyte lacunae (*59*–*61*). In contrast, the *Bothriolepis* lacunae differed, from traditional spindle-shaped lacunae, to small round lacunae (fig. S2). The morphological and size variation of cell lacunae within this close proximity is previously undocumented in fossils, likely due to insufficient resolution in imaging technology [although see (*62*)]. The smaller lacunae were often rounder in shape, although this was not quantified; the size and morphological variation in this sample is notably extreme and unusual (figs. S2 and S3). Osteocyte shape is an important measure, as it is relevant to their mechano-sensing function (*58*, *63*), and some studies claim that particular osteocyte shapes are more sensitive than others (*64*). Osteocyte shape may also be related to bone type, with lacunae in organized (e.g., lamellar or parallel-fibered) bone being more elongate and regularly shaped than in woven bone (*21*, *65*). On the other hand, the large disparity in osteocyte size is relevant to paleontological genome estimation (*5*, *6*, *21*, *66*). Here, we consider even the smallest segmented spaces to be true biological structures that would have housed cells and/or their processes; this is due to the following criteria: The shape of the spaces is organic and lacks hard edges that would have been created by crystallization. The smallest spaces always have branching canaliculi emanating from them and connecting to nearby cells; these smallest structures could be enlarged canalicular junctions as characterized by a recent study by Wittig *et al.* (*67*). In the Wittig study, the canalicular junctions were around 30 μm^3^ in volume; however, imposing such a restriction on our dataset is arbitrary as the volume of the spaces is continuous. A possible explanation could be due to enlarged junctions and small osteocyte lacunae having overlapping sizes. Measurements at this scale are still rare, whether it be on fossils or modern taxa; therefore, the authors believe that further studies may help in distinguishing junctions from true lacunae in the future. Last, the authors are unaware of any other physiological structure or taphonomic process that would result in a clustering of small spaces with branching connections and therefore are confident that these are biological structures.

The most novel data, however, are those of the canalicular processes, which have previously only been imaged in 2D in fossil material (*16*, *17*) and only captured in 3D as noncontinuous fragments in modern material (*21*). Imaging and quantifying of the LCN in 3D have been challenging even in modern specimens effectively being done for the first time in mice (*54*). Goggin *et al.* (*60*) concluded that serial FIB-SEM imaging was the only true way to quantify the morphology of the LCN, and later studies agree that serial FIB-SEM produced the highest-resolution 3D tomography. Our FIB-SEM data have shown that larger cell lacunae have more canaliculi emanating from them, and in the *Bothriolepis* sample, the canaliculi have a bidirectional bias that is almost perpendicular (Fig. 4C). This is reminiscent of osteocytes in lamellar type bone in humans (*56*). The FIB-SEM resolution also allowed the analysis of canalicular branching; the canaliculi of the *Bothriolepis* sample was shown to branch up to five times (Fig. 5D), whereas the *Tremataspis* sample only branched three times (however, the latter is likely biased by the smaller field of view and a smaller sample size). The *Bothriolepis* sample branching up to five times is the same number of branching that has been recorded in humans (*2*). Whether this means that osteocyte canaliculi inherently only branch up to five times in gnathostomes is impossible to determine, as this analysis has only previously been done on human osteocytes (*2*), and therefore, we are limited in our comparisons.

### Osteocytic osteolysis and evolutionary implications

Osteocytic osteolysis is one of the focuses of this study, as it is a physiological process inherently absent from the earliest anosteocytic bone and therefore represents a potential advantage of osteocytic bone. In the sampled osteostracan specimen, there are areas interpreted as regions of low density that are only found around osteocyte lacunae and some canaliculi (Fig. 4). The areas of low density were not equally distributed, and they were also not found in the placoderm specimen. These areas of low density are not randomly arranged, nor do they appear in every hollow space sampled, and therefore, we conclude that they are not a result of taphonomy but rather are evidence of a physiological process that occurred in life. We interpret this as evidence of mineral metabolism via osteocytic osteolysis.

Osteocytic osteolysis is the dissolution of bone mineral by osteocytes. This is done through ion pumps that decrease the pH of the fluid within the lacunae and canaliculi, allowing the dissolution of minerals from the peri-lacunar walls (*11*, *12*, *45*, *68*–*71*).

Osteocytic osteolysis was initially a contentious issue because of the conflation of this phenomenon with the now disproven bone flow theory (*72*), and the initial conclusion that osteocytic osteolysis was the principal means of bone remodeling (*44*). Many experiments have since shown that osteocytes are capable of osteolytic activity but are not the main agent of remodeling of bone tissue, which is primarily performed by osteoclasts (*30*, *73*). A recent study by Nango *et al.* (*69*) has shown that demineralization can be detected along the canaliculi in synchrotron μCT images of mice tibiae.

Modern anosteocytic bone has been shown to remodel by mononucleated osteoclasts (*30*, *41*–*43*), and it is reasonable to assume that osteoclasts were present alongside the first bone tissue as there is evidence of resorption in both acellular and cellular bone of early jawless vertebrates (*16*, *37*, *39*, *74*–*76*). The first definite evidence of organized osteonal remodeling that is led by osteoclasts does not appear until early gnathostomes, the antiarch placoderms who have the earliest record of remodeling via secondary osteons (*28*, *37*). Therefore, osteoclasts likely existed in the early osteostracans as evidenced by surface bone resorption, but internal bone resorption in the form of secondary osteons made by osteoclasts is only known in gnathostomes (the earliest occurrence being found in antiarch placoderms). Simply put, osteoclasts existed but one of their main modern functions did not. Therefore, one should not assume that, just because there is some evidence of the existence of a cell type, it necessarily performed the same functions as in their modern counterparts. Similarly, just because osteocytic bone appears in the fossil record, it cannot be simply assumed that the first osteocytes held all the functions of modern osteocytes (in this particular case, the function of osteocytic osteolysis).

In mammals, osteocytic osteolysis occurs pathologically or in times of calcium deficiency. However, in teleost fishes, calcium is often readily available in the environment, and phosphate is the limiting factor (*46*, *77*). In teleosts with cellular bone, osteocytic osteolysis has been shown to occur before and during migration (*60*), likely linked to an increased demand for metabolic calcium or phosphate requirements. In addition, osteocytes have been acquired secondarily in some endothermic teleosts (tunas and opahs) from anosteocytic ancestors, potentially also due to increased metabolic needs (*31*, *78*, *79*). Because most jawless vertebrates lived in marine environments, we assume that calcium was readily available to them, and if osteolysis was occurring for a metabolic reason, it was likely due to a lack of phosphate similar to the case in modern fishes (*77*, *80*). In other jawless vertebrates, such as heterostracans with anosteocytic bone, osteocytic osteolysis is inherently impossible, making it much more difficult to mobilize mineral storage as they would have to rely on osteoclasts at the bone surface. Therefore, if osteostracans could use the surface area of the osteocyte LCN in their skeleton (which is several orders of magnitude greater than the area of the bone surface for mineral storage), and because this study shows the osteolytic mechanism by which they could release and restore this mineral, then the presence of osteocytes would provide a strong advantage over the anosteocytic bone of temporally co-occurring heterostracans. This metabolic advantage could then explain why all vertebrate life maintained osteocytic bone, until its secondary loss within teleosts.

## CONCLUSIONS

Here, we show that osteocytic osteolysis was a likely mechanism for mineral metabolism used by the earliest osteocytic bone. We suggest that this process linked to the physiological demand for phosphorus was one of the principal drivers in the initial evolution of osteocytic bone. Mineral metabolism performed by osteocytic osteolysis then provided an undoubtable advantage to osteocytic over anosteocytic vertebrates. This advantage was possibly so profound as to alter vertebrate evolution, as jawed vertebrates (to the notable exception of more than half of teleost fishes) retained osteocytic bone. FIB-SEM tomography, a newly integrated technology allowing high-resolution imaging of fossilized cell spaces, was used in this study to study fossilized bone. We anticipate that this method can be used to help differentiate and characterize tissues in the earliest mineralizing vertebrates and other fossil organisms.

## MATERIALS AND METHODS

### Histology

Histological sections were obtained from W. Gross’s historical thin section collection at the Museum für Naturekunde, Berlin. Therefore, the thin sections imaged here were not made for this study but were only reimaged for this study. Last, we rely on W. Gross’s labeling of these thin sections that often did not include a species name, as often only fragments were sectioned.

### Sample preparation

The same preparation techniques were applied to two fossil samples, i.e., the osteostracan *Tremataspis mammillata* (MB.f.9025) from the Late Silurian (~423 to 428 million years ago) of Saaremaa, Estonia, and the antiarch placoderm *Bothriolepis trautscholdi* (MB.f.9188a) from the Late Devonian (Frasnian, ~372 to 382 million years ago) of the banks of the Syas River near the village of Stolbovo, Russia. At first, the fossilized bones were cut with a diamond wire saw (well Diamantdrahtsägen GmbH) with a 0.2-mm wire diameter. The resulting 10-mm^3^ blocks were then embedded under vacuum condition using EpoThin2 with a resin to hardener ratio of 20:9. After 1 day of curing, the samples were grinded and polished, first mechanically and then via argon ion milling. As the last preparation step, the samples were fixed on a SEM aluminum holder using conductive silver glue and finally a gold layer was sputtered on top to ensure surface conductivity.

Large-scale SEM mappings have been recorded to localize areas with a high chance for successful FIB topographies as shown in fig. S1. Areas with inclusion cracks or without signs of cell pores on the surface were not used.

### FIB-SEM tomography

FIB-SEM tomography was carried out using ZEISS Crossbeam 340. During tomography, a gallium ion beam sequentially sputters (or grinds) away sample material by precisely targeting the sample with focused gallium ions at 30 keV and 3 nA. As the gallium gun (thus, the gallium beam) is angled at 54° in relation to the electron gun, it is possible to scan the sputtered edge of the sample with the electron microscope at the same time as the edge is further sputtered away. This technique is called serial sectioning, and it allows a very precise 3D scanning of samples with a resulting voxel size below 10 nm^3^. To reduce local charging induced image artefacts, the fossilized samples were measured with an energy-selective backscattered InLens detector. The electron aperture was set to 120 nm. The voxel size was 20 nm for the *Tremataspis* sample and 30 nm for the *Bothriolepis* sample. In both cases, the electron cycle time was 20 s and the ion cycle time was 15 s. For the *Tremataspis* sample, a 33 × 36 × 23 μm^3^ volume of usable data (excluding undefined volume borders) was measured over a period of 11 hours. For the *Bothriolepis* sample, a 56 × 41 × 50.0 μm^3^ volume of usable data was measured over a period of 18 hours.

### Data processing and analysis

If not stated otherwise, all image calculations have been done by using ImageJ (*81*). The raw data captured with FIB-SEM were first drift corrected using algorithms based on SIFT (scale invariant feature transform) (*82*). Then, the image border areas containing information with incomplete milled material had to be removed from the dataset, rendering them undefined. The gray values of those voxels in those undefined areas have been set to the gray values of the closest defined voxels. As the epoxy-filled cavities inside the bone are much darker than the mineralized bone itself, they can be virtually filled with a morphological closing operation. The resulting stack was then gaussian-filtered and was subtracted from the original drift corrected data to normalize the gray values of the dataset. At last, the noise of the image stack has been reduced by applying a nonlocal means filter with a photometric distance set to the standard derivation of the milled bone surface. The *Tremataspis* dataset contained a horizontal crack with a diameter of about 100 nm through the whole volume that was morphologically segmented by means of a directionality analysis and filled up virtually. Final binarization of the *Tremataspis* dataset was done by thresholding the normalized dataset. To reduce the noise in the binary dataset, a connected component analysis has been carried out, removing not connected very small pore artefacts. In the *Tremataspis* dataset, a third phase was discovered surrounding three former pore structures; that phase was manually segmented.

The *Bothriolepis* dataset contained a large T-like crack structure dividing the dataset in three pieces, resulting in fault artefacts. To remove the crack and for fault artefact correction, the three separated pieces of the dataset were segmented individually and virtually shifted back to their original position. The classification of the pore space of the *Bothriolepis* dataset was not possible with a threshold-based procedure, as the pore system was closed and thus not filled by resin. For the pore classification, a convolutional neural network autoencoder based on a 3D U-Net structure was used (*83*). Although the autoencoder was originally trained on battery material, it was able to detect the *Bothriolepis* pore space so that no manual classification was needed. The segmented datasets were then analyzed with the MorpholibJ plugin (*84*). For the separation of canaliculi and cell pores, the local thickness plugin that is integrated in ImageJ was used. After the separation, the individual parameters such as total cell count or canaliculi diameters were measured with MorpholibJ. If not stated otherwise, in the following, data fitting and plotting were performed using OriginPro 2016 (*85*). For cell diameter analyses (Fig. 5B and fig. S1E), border cell lacunae were removed. Then, a frequency count analysis was conducted on the cell diameter values with a binning number of 6. A normal distribution was then fitted into the *Bothriolepis* histogram using Origins “Gauss” function (table S2B). Because of the low number of detected cell lacunae in the *Tremataspis* data, we did not apply a fitting function on this dataset.

For the pore network analysis, the canaliculi have been first skeletonized. Second, the path junction voxels have been expanded using a maximum filter, resulting in a dataset with the canaliculi reduced in its diameters to one voxel and the junctions now represented by small spherical pores with a diameter of three voxels. After that, a watershed transformation was applied to cut the reduced canaliculi in the middle between two neighboring junctions. The junctions now represent little separated quasi-pores themselves, and the canaliculi still contain the information where junctions are connected with each other. Combined with the original cell pores, that dataset was then transformed and measured using PoreSpy (*86*) and OpenPNM (*87*). The resulting network graph contained the labeled pores, and its connections combined with its parameters: mainly its diameters, positions, direction, and the information about which objects are connected with each other. For the correlation analysis shown in Fig. 5B, the number of outgoing canaliculi per cell was plotted over the individual cell diameters and a linear regression model was applied. The segmentation of the canaliculi into section classes representing how many junctions the sections were away from a cell space was directly extracted from the network graph and plotted (Fig. 6C and fig. S1, A, C, D, and F). For the canaliculi section directionality analysis in Fig. 5C, a 2D frequency count analysis was applied over the channel section angles θ and φ (spherical coordinate system) with binning numbers of 90 × 90. From the resulting network graph, the canaliculi branching behavior was extracted and visualized using Paraview (*88*) and Blender 3D.

For the canaliculi diameter analysis, the previously generated local thickness data were used by multiplying it with a skeleton mask of the canaliculi network. The result was denoised with a median filter (radius, 150 nm). The median was necessary for data points at little grains inside the canaliculi that, if not filtered, resulted in unrealistic bottlenecks. Each voxel representing a canaliculi section was then counted and plotted. For a better comparison, both histograms have been rescaled so that the sum of the values of each dataset would be equal (100%). Logarithmic normal distributions have then been fitted into the equalized histograms using Origins “LogNormal” function (table S1A).

### Limitations

This new application of FIB-SEM tomography on fossil material provides valuable new information on the morphology and interconnectivity of the earliest osteocytes and their processes by proxy of their lacunae and canaliculi, but it is equally important to understand what cannot be deduced from this methodology. For example, due to sampling restrictions, the areas that would most likely yield a high number of lacunae were sampled, and therefore, osteocyte density analysis is prebiased by the method of sampling and is not a reliable estimate of the entire sample. Another limitation is the field of view. Because of limited field of view, even if a lacuna is captured in its entirety, the entire length of the outgoing canaliculi often cannot be measured because they often continue outside the field of view. This renders any estimates of interconnectivity a gross underestimation. The limitation of the field of view is exemplified in the number of measured lacunae in the *Tremataspis* specimen, which was our first measurement and resulted in far fewer cell spaces than our second measurement. The smaller number of cell spaces results in a broad confidence band (Fig. 5B and table S2A).

## SUPPLEMENTARY MATERIALS

Supplementary material for this article is available at http://advances.sciencemag.org/cgi/content/full/7/14/eabb9113/DC1

## Appendix

View/request a protocol for this paper from *Bio-protocol*.
